# IgG4-Related Pericarditis Diagnosed by Accumulated Pericardial Effusion

**DOI:** 10.1155/2023/9223342

**Published:** 2023-11-24

**Authors:** Hirohito Sugawara, Tomokazu Takahashi, Yukishige Kimura, Azumi Matsui, Tamaki Matsumoto, Kimio Nishisato, Mitsuhiro Nishimura

**Affiliations:** ^1^Department of Cardiology, Tenshi Hospital, 1-1 North-12 East-3, Higashi-ku, Sapporo, Hokkaido, Japan 0658611; ^2^Division of Nephrology, Department of Internal Medicine, Showa University Northern Yokohama Hospital, 35-1, Chigasakichuo, Tsuzuki-ku, Yokohama, Japan 2248503

## Abstract

IgG4-related pericarditis has rarely been reported. Here, we report a case of IgG4-related disease that presented with pericardial effusion. A 67-year-old female who presented with palpitations and chest pain was admitted because of a large amount of pericardial effusion that required drainage. The patient underwent pericardial drainage, and the symptoms were gradually alleviated. IgG4 levels were elevated in the serum and pericardial effusions. A biopsy specimen of ^18^F-FDG accumulated in the submandibular gland showed lymphocyte infiltration with IgG4-positive cells. The patient was diagnosed with IgG4-related pericarditis. Glucocorticoids resolved serological and imaging abnormalities. Prompt treatment improves the disease status.

## 1. Introduction

IgG4-realated disease (IgG4-RD) is a relatively rare autoimmune disorder associated with elevated serum IgG4 concentration, lymphoplasmacytic infiltration of IgG4-positive plasma cells, and storiform fibrosis in various organs. Here, we present a case of IgG4-RD presenting with pericardial effusion.

## 2. Case Presentation

**Figure 1:**
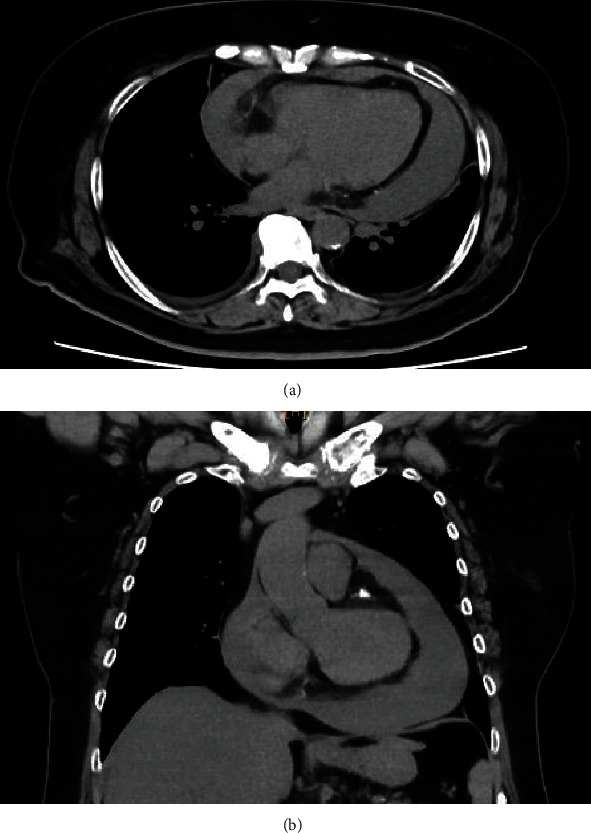
The findings of pericardial effusion before drainage and steroid therapy in computed tomography (CT) (a, b).

A 67-year-old female presented to our hospital with palpitations and chest pain. The patient had a history of asthma and diabetes mellitus (DM). Computed tomography (CT) revealed a large amount of pericardial effusion, and the patient was admitted (Figure 1).

**Figure 2:**
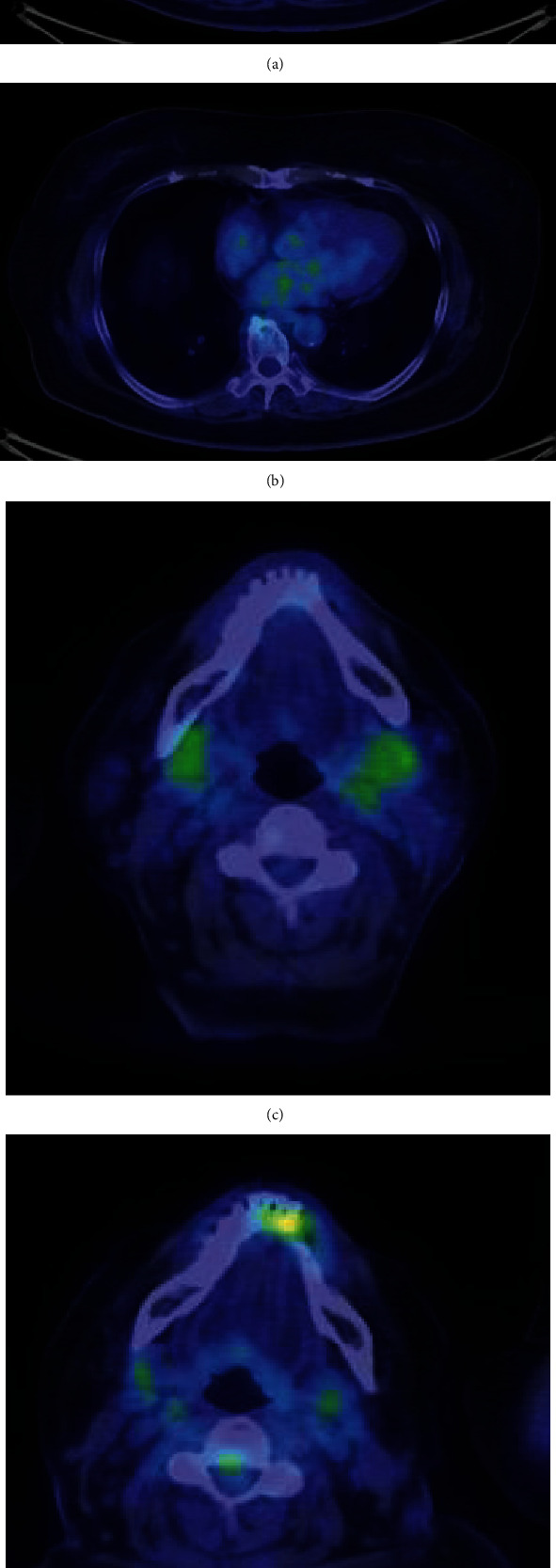
Positron emission tomography (PET)/CT images before and after steroid therapy. Abnormal ^18^F-FDG uptake was found in the pericardium (a) and submandibular gland (c) before treatment. After treatment, ^18^F-FDG uptake in the pericardium (b) and submandibular gland (d) is diminished.

**Figure 3:**
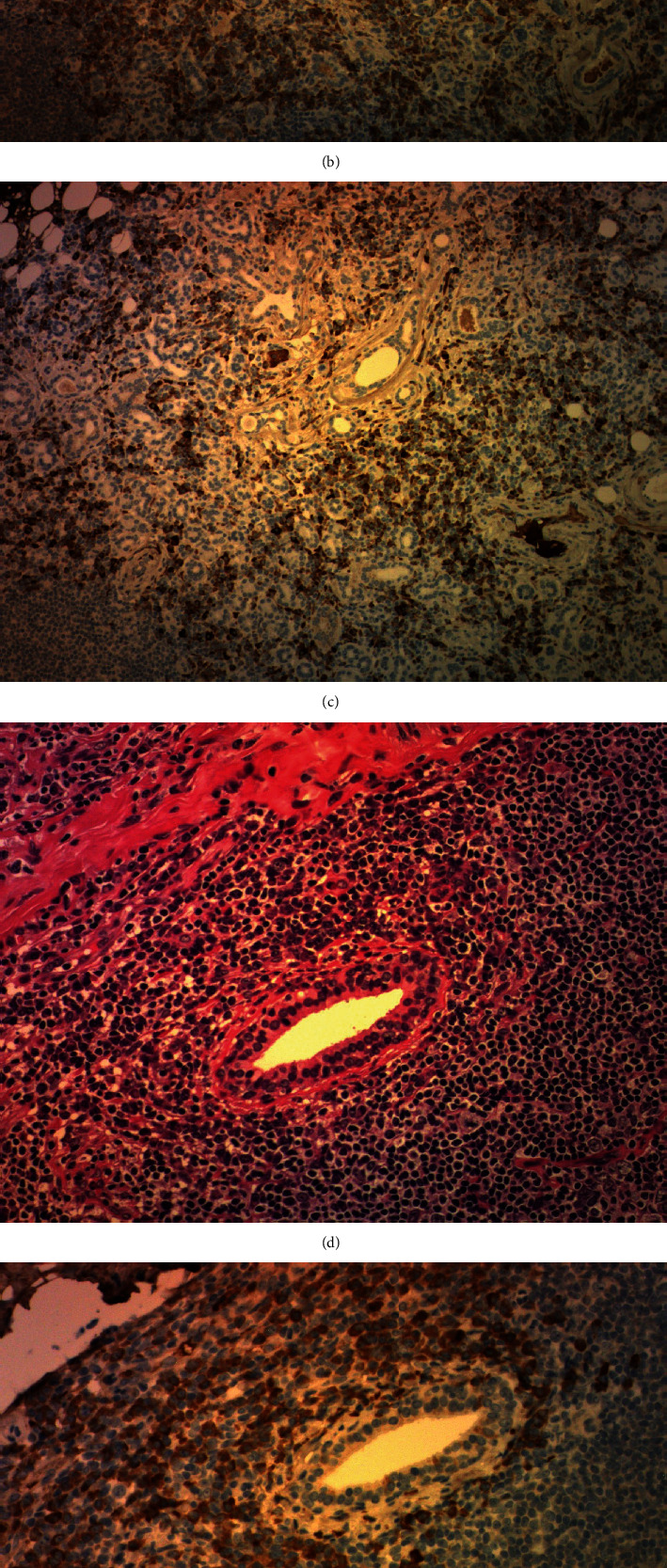
Histological sections of biopsy specimens from the submandibular gland. Lymphocytic and plasma cell infiltration (a (100x), d (200x)). Most of the inflammatory cells were positive for IgG immunohistochemical staining (b (100x), e (200x)). More than 40% of the IgG-positive cells were reactive to IgG4 immunohistochemical staining (c (100x), f (200x)).

On admission, her blood pressure was 100/70 mmHg and had a regular sinus rhythm with a rate of 100 beats per minute. Her heart and lung sounds were clear, and lower leg edema was not observed. The laboratory tests showed white blood cells of 7,520/*μ*L, eosinophil of 790/*μ*L, total protein concentration of 9.3 mg/dL, C-reactive protein (CRP) concentration of 1.53 mg/dL, and N-terminal probrain natriuretic peptide (NT-proBNP) of 223 pg/mL. IgG and IgG4 levels were markedly elevated in the serum (3,850 mg/dL and 2,281 mg/dL, respectively) and pericardial fluid (4,898 mg/dL and 3,761 mg/dL, respectively). The IgE levels were also elevated (271 IU/mL). Elevations in viral antibody and tumor marker levels and serum autoantibodies related to collagen diseases were not observed. Electrocardiogram revealed a sinus tachycardia. The patient underwent pericardial drainage, and her symptoms were gradually alleviated. Positron emission tomography (PET)/CT revealed ^18^F-fluorodeoxyglucose (^18^F-FDG) uptake in the pericardium and submandibular gland (Figures 2(a) and 2(c)). Histological and immunohistochemical examination of the biopsy specimen of ^18^F-FDG accumulated in the submandibular gland showed an infiltration of lymphocytes with IgG4-positive cells (10 per high-power field) and an IgG4-positive/IgG-positive cell ratio of 40% (Figure 3). Based on these findings, we diagnosed the patient with IgG4-related pericarditis according to the comprehensive diagnostic criteria for IgG4-RD.

Oral prednisolone was administered at a dose of 50 mg daily, and the serum IgG4 level gradually improved to 235 mg/dL. Follow-up PET/CT showed disappearance of ^18^F-FDG uptake in the pericardium and submandibular gland (Figures 2(b) and 2(d)). Disease recurrence and symptoms of heart failure were not observed during the year follow-up.

## 3. Discussion

IgG4-RD is a multiorgan disorder characterized by the infiltration of IgG4-positive plasma cells in various organs with a high level of serum IgG4 [1]. The disorder was first reported in 2001 in patients with autoimmune pancreatitis [2]. Involvement of the pericardium is rare, but a growing number of cases have been reported. Doumen et al. summarized cases of IgG4-RD with pericardial involvement. In a total of 33 cases, the sole manifestation of IgG4 pericarditis was small, and most cases (17 cases, 52%) have been reported in Japan [3]. IgG4-RD is a novel disease concept introduced in Japan. Therefore, it is possible that the disease itself is more prevalent in Japan or that the disease is not well understood, so it is an undiagnosed case worldwide.

The comprehensive diagnostic criteria for IgG4-RD revised in 2020 were as follows: (1) one or more organs showing diffuse or localized swelling or a mass or nodule characteristic of IgG4-RD, (2) serum IgG4 levels greater than 135 mg/dL, and (3) positivity for two of the following three criteria: (1) dense lymphocyte and plasma cell infiltration with fibrosis, (2) ratio of IgG4-positive plasma cells/IgG-positive cells greater than 40% and the number of IgG4-positive plasma cells greater than 10 per high-power field, and (3) typical tissue fibrosis, particularly storiform fibrosis, or obliterative phlebitis [4]. In the diagnostic criteria, biopsy specimen is essential for making a definitive and probable diagnosis. IgG4-related pericarditis is difficult to diagnose histologically [5]. PET/CT was performed to determine the biopsy sites. IgG4-RD involves a variety of pathological changes that are dependent on the tissues involved; therefore, this modality would be helpful [6]. Horie et al. performed a cytological examination of pericardial effusion [7]. The IgG4 level in the pericardial effusion, as in our case, may support the diagnosis of IgG4-related pericarditis. In the present case, all these conditions, including PET/CT imaging and pathologic examinations showing characteristic changes and elevated serum IgG4 levels, led to a definitive diagnosis of IgG4-related pericarditis.

Most patients with IgG4-related pericarditis show a rapid clinical response to steroid therapy [8, 9] and which may prevent progressive involvement of other organs [10]. However, delayed steroid therapy may lead to constrictive pericarditis. Constrictive pericarditis occurs in approximately 2-3% of cases [11]. Therefore, prompt therapy may suppress the progression to constrictive pericarditis.

## 4. Conclusion

The diagnosis of IgG4-related pericarditis is often difficult based on histological analysis. PET/CT helps identify IgG4-related organs and is useful for evaluating the efficacy of steroid therapy for IgG4-related diseases.
